# Determinants of Complete Immunization Coverage among Children Aged 11-24 Months in Somalia

**DOI:** 10.1155/2020/5827074

**Published:** 2020-05-15

**Authors:** Abdinasir Abdullahi Jama

**Affiliations:** Department of Obstetrics & Gynecology, Pan African University/University of Ibadan Nigeria, University College Hospital (UCH), University of Ibadan, Nigeria

## Abstract

**Background:**

The general coverage of vaccination means the percentage of children in world who received the recommended vaccines and has existed over the past few years continued the same.

**Aim:**

The aim of this study was to determine the factors associated with complete immunization coverage among children aged 11-24 months in Somalia.

**Method:**

This was a descriptive cross-sectional study conducted in Galkayo hospital in Mudug region Somalia. Systematic random sampling was carried out in to recruit 357 children between 24 and 11 ages. Well-structured questionnaires were filled by participants who gave their consent. Data were analyzed using the statistical package for social sciences version 21. Descriptive statistics such as means, standard deviation, proportions, and range were used to summarize the data. Inferential statistics were used to test for association between the dependent variable and independent variables using the chi-square with the level of significance set at 5%.

**Result:**

The age of the respondents was between 11 and 24 with a mean age of the 7.71 with standard deviation ±5.87, the level of education of the mother (*p* = 0.0001), the place of delivery of the baby (*p* = 0.0001), and the distance of the participants to health facility (*p* = 0.026533) were statistically significantly associated with immunization coverage. Final the full immunization coverage in Somalia is 20%.

**Conclusion:**

The study is recommended to the government to increase the level of education of the mother; also, the study is recommended to increase the hospital delivery that may increase the immunization coverage in Somalia.

## 1. Background Information

The general coverage of vaccination means the percentage of children in world who received the recommended vaccines and has existed over the past few years continued the same. In 2017, almost 80% of infants in the world got three doses of DPT3 to protect the serious diseases that can result in illness and disability or even death to the children. Also in this year of 2017, the 123 countries had reached at least 90% of the DPT3 vaccine [1].

The Expanded Program on Immunization (EPI) look for to reduce morbidity and mortality of children by giving vaccine against to words the preventable disease like diphtheria, pertussis, tetanus, measles, poliomyelitis, and tuberculosis, and it is important to the children in the world especially those in the developing countries. The program needs the effective implementation and evaluation of the program outcome, objectives, and its target [2]. There is research indicating that at 24 months of age 42% of Latin children and 26% of Africans were immunized [3]. The number of case of the measles immunization program in the developed country has reduced due to the vaccine expanded program [4]. But the measles is still remaining the major health issue in developing countries especially the remote and urban area. For example, Zimbabwe, although there was a recent achievement of the past few years, the measles is endemic with transmission peaks between August and December [5].

In Pakistan, the research has found that EPI despite 20 years of struggle could not establish an organized information/motivation program for crowds and to educate them on the importance of child immunization. Moreover, a doubt in the mind of mothers about the nonavailability of vaccine or the absence of vaccinator at the place of immunization was also an important factor in the failure of the immunization program. All this shows the inefficiency of the program and requires a serious thought by the EPI department. The other important aspect revealed that the researcher was parents/caretakers' perception regarding the importance of the immunization card. He found only 36% of mothers could produce the immunization card in support of their child's immunization status. This showed that the immunization card was not considered an important document [6]. Also, the immunization coverage in India is far from complete with a disproportionately large number of rural children not being immunized [7].

In the past decade, the vaccination coverage has been improved in Ethiopia, the incidence of measles has increased from 3.19/100,000 in 2009 to 7.35/100,000 in 2010. The Ethiopian Demographic and Health Survey (DHS) 2005 revealed that only 20% of children 12–24 months of age were fully vaccinated, and 24% of children did not receive any vaccination. Children were more likely to be vaccinated the first doses of vaccination than the third and the fourth doses in which 60% of children received BCG and from this only 35% of them received measles vaccine [8].

Mukungwa [9] found out that birth order was an important predictor of full immunization in Zimbabwe. This is likely so because for the lower birth order children, mothers are enthusiastic about having children, and they exert appropriate care and upbringing of the children.

WHO and health partners support immunization activities in approximately 200 fixed centres and outreach vaccination sites across the country. WHO has established and provided support to immunization units within ministries of health and is instrumental in supervising and coordinating immunization activities in the northern regions of Somalia. WHO continues to train health workers, improve cold chain, and support supervisory and monitoring activities [10].

The researches done in Zimbabwe were found that full vaccination among children aged 12-23 months in Zimbabwe is determined by the region of residence, wealth status, birth order, place of delivery, antenatal care during pregnancy, and exposure to television are significant [9].

## 2. Issue of This Case in Somalia

Despite this, only 30–40% of children are immunized against the six major childhood diseases. This is relatively low compared to the global coverage of almost 80%. Routine child immunization coverage among one-year-old children for measles is 24% and for diphtheria, tetanus, and pertussis (DTP3) is 31% [11].

Control of vaccine-preventable diseases remains a huge challenge in Somalia, due to the low routine immunization coverage and the continued inability to reach almost 600 000 under-five children with supplementary immunization activities. The polio outbreak that hit Somalia in May 2013 is a strong reminder of the risks posed by a large cohort of unimmunized children, as of the end of February, the total number of polio cases in Somalia stands at 194. The two most recent cases had the onset of paralysis on 20 December 2013 and were reported in Somalia [12].

The determinant of this issue is unknown in Somalia that is why they mostly happen in a large area in Somalia.

Despite the report from the WHO or UNICEF, there is no single research that has been done in this issue for factors determined by the immunization coverage of children less 2 years in Galkayo, Somalia, but there are only a few researchers done in Somalis in Ethiopian region that indicated low coverage immunization of children less than 2 years; thus, this study will focus on the immunization coverage of children less than 2 years. In general, the immunization coverage in Somalia is unknown except for some reports from WHO or UNICEF; these organizations have estimate immunization coverage in Somalia. The study will form a basis for future discussions and research in the area of vaccination to facilitate the adoption of better strategies to improve access and to know how many have got the immunization to exist in Galkayo, Somalia. This study was based on to answer the following questions:

- What are the determinants of immunization coverage among 11-24 months in south Galkayo district?
- What is the prevalence of immunization coverage among 11-24 months in south Galkayo districts?

## 3. Materials and Methods

### 3.1. Study Site

This study was conducted in Galkayo public hospital, Mudug Galkayo, Somalia. Somalia is a country located in the Horn of Africa. It is bordered by Ethiopia to the west, Djibouti to the northwest, the Gulf of Aden to the north, the Indian Ocean to the east, and Kenya to the southwest.

Galkayo Hospital began his building at the end of 1999 by community members, business persons, diasporas, and intellectuals. This building was organized by Mudug Development Organization (MDO). The hospital began its work in April 2000 for emergency and OPD consultation and inpatient departments. It is the only public hospital in Galkayo so that it receives the highest number of patients in Galkayo.

The number of the children who visited the hospital per months is 6000 according to data the researcher got in 2018.

### 3.2. Study Design

The design of the study will be a descriptive, cross-sectional study to investigate assessment factors associated with complete immunization coverage among children in aged 11-24 months in Galkayo public hospital.

### 3.3. Inclusion/Exclusion Criteria

This was only focused on the children 11 to 24months age group who visited at Galkayo public hospital without considering their health status. Also, it was excluded if they had poor caretakers who cannot give information of his/her child. And those who denied to participate our research.

### 3.4. Sample Size Determination

The sample size was calculated using the formula of n = (*z ***α**)^2^ *p* × q /(d)^2^ (Leslie kish Formula), with 36.6% of prevalence found the research conducted the Somalis region in Ethiopian [13].

Where

(1) $$\begin{array}{c} \;n=\text{the desired sample size};\\ Z\alpha =\text{standard normal deviate at }95\%\text{confidence limit}=1.96^{\prime };\\ p=\text{maximum variability of the population};\\ q=\text{the difference between }1\text{ and }p;\\ d=\text{is the desired level of precision }\left(\text{i}.\text{e}.,\text{the margin of error}\right).\\ \text{Finally},\text{it was founded}=n=\frac{{\left(1.96\right)}^{2}0.36.6\times 0.63.4}{{\left(0.05\right)}^{2}}=357 \end{array}$$

A systematic random sampling probability technique was carried out in hospitals to conduct the study. First, the start number/sampling interval (*K*) that was 16 was calculated from the children visited at Galkayo hospital then chose the start number by lottery methods, and the start number was 5 then was continued this interval until the desired number was achieved.

(2) $$\begin{array}{c} K:\frac{6000}{357}=16 \end{array}$$

### 3.5. Data Collection Procedure

The study used a well-structured questionnaire which was drafted from literature. The objectives of the study were well explained to take care, and those who agreed and gave written consent were made to fill the questionnaire. The questions were administratively administered, so the researcher was filling out of the questionnaires from the participants. Because Galkayo hospital has three work shifts for medical personnel—morning, afternoon, and night; the collection of data was planned during the morning shift where the most OPD patients come to the hospital. Data collection spanned between the period of May 2019 to June 2019.

### 3.6. Data Analysis

Collected data were verified, coded, and summarized, before they were analyzed using the IBM SPSS version 20.0 computer software. Descriptive statistics of frequencies, percentages, mean, and standard deviations were generated. Statistical inferences were tested using the chi-square method with a level of significance set at *p* < 0.05.

### 3.7. Ethical Consideration

The study was thought about the ethical issues throughout research and will keep the privacy and confidentiality of the respondents from the hospital. Every respondent was asked for permission to complete the questionnaire. The ethical approval will be getting from Galkayo University and the administrative of Galkayo Hospital.

## 4. Results

Table 1 shows the sociodemographic characteristics of the participants. The age range of the children in the study was 11-24 months with a mean of 7.71 ± 5.87. The majority (73.7%) of the mothers in the study have nonformal education level; also, the majority of them was married and nonemployed status. The family income of the majority of the participants was between 160-210 and 220-600. The majority (31.1%) of the participants were living in Howl/Wadag; most of the respondent (53.5%) reported that they have not visit ANC during their pregnancy of this child. The majority them have one to four child, and finally most of them has female child.

## 5. Immunization Coverage in Somalia

**Figure 1:**
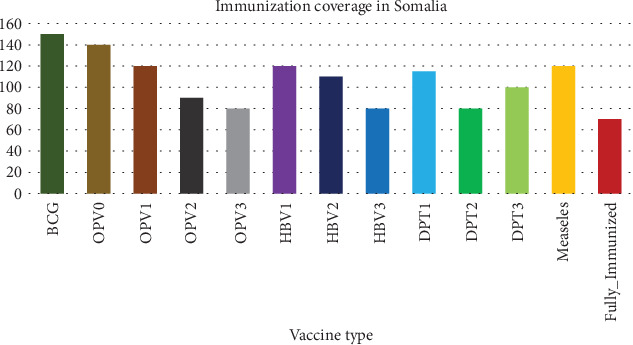
The immunization coverage of investigated children (11-24 months) in Somalia.

Figure 1 is showing the immunization coverage in Somalia over the 357 participants we interviewed, and it was found that 150 (42%) of them had taken Bacillus calmette. Guerin vaccine (BCG vaccine) and oral polio vaccine (OPV) status of the participants was 140 (39%) have taken OPV 0, 120 (33%) have taken OPV 1, 90 (25%) have taken OPV 2, and finally 80 (22%) have taken OPV 3. Hepatitis B vaccine (HBV) status of the participant was 120 (33%) of them had taken HBV 1, 110 (30%) had taken HBV2, and 80 (22%) of them had taken HBV3. The diphtheria, pertussis, and tetanus status of the participant were found that 115 (32%) had taken DPT1, 80 (22%) have taken DPT2, and 100 (28%) of them have taken DPT3. Figure 1 shows the measles vaccine of the participants as 120 (33%) of them have taken the measles vaccine. The final figure is showing that 70 of them were fully immunized over 357 of the participants, which makes 20% of immunization coverage.

## 6. Factor Immunization Coverage in Somalia

As part of the study objectives, possible factors associated with immunization coverage was determined using the chi-square. The results are shown in Table 2. Results showed a significant association between immunization coverage among the children and the level of education of the mother (*p* = 0.0001), the place of delivery of the baby (*p* = 0.0001), and the distance of the participants to health facility (*p* = 0.026533) were statistically significantly associated with immunization coverage.

Other factors found not to be significantly associated with immunization coverage were the occupation of the mother, age of the baby, sex of child, marital status of the mother, antenatal care for the mother, place of residence of the participants, and the birth order of the baby with *p* < 0.05.

The predictors in this study that indicators as the immunization coverage factors are the place of delivery of the baby, level of education of the mother, and distance to health facilities.

The place of delivery of the baby is influence whether the child has full immunization or not, and this study shows that the child was delivered at the hospital and was 7.3 more likely to have full immunization compare to the child who delivered at home. In the level of education of mothers were also associated the full immunization of the child as this study presented, the child from the mother with primary level of education was 18 times more likely to have full immunization than the child from the mother with nonformal education. Also, the child whose mother had secondary education were 10 more likely to have full immunization, than the child whose mother had nonformal education; the child whose mother had tertiary education also 1.8 times more likely to have full immunization, than the child whose mother had no formal education.

Finally, the study shows that there is no significant association between the distance to health facilities and the full immunization coverage of the children between 11 and 24 years.

## 7. Discussion

The study focused on the factors associated with complete immunization coverage among children aged 12-23 months in Galkayo public hospital. A cross-sectional survey method was used to recruit participants who are aged 12-23 months and their mothers with currently at OPD service at the study sites.

The study revealed that the level of education of the mother showed a statistically significant association with the full immunization coverage in Somalia. Similar secondary analysis of cross-sectional survey data conducted in the Sindh province of Pakistan found a similar result [14].

The study also showed that the place of delivery of the baby was a statistically significant association with the immunization coverage that means the babies who were delivered at hospital were most likely to get full immunization coverage, while the babies born at home were less likely to get full immunization coverage. Similar studies [13, 15] conducted in Jigjiga District, Somali National Regional State, Ethiopia, and Debre Markos Town, Amhara Regional State, Ethiopia, respectively, showed similar results.

Further analysis from the 2016 Ethiopia concerning demographic and health survey found 38.0% as full immunization coverage [16], while another study conducted in factors in Jigjiga District, Somali National Regional State, Ethiopia, found 36.6% as full immunization coverage [13].

## 8. Conclusion

The study found out that the immunization coverage in Somalia is influenced. The level of education of the mother is associated with the immunization coverage of their child, the place of delivery of the baby (home or hospital) is associated with the immunization coverage of the child, and the distance of the participants to a health facility is associated with the immunization coverage of Somalia. The full immunization coverage in Somalia is 20%.

## 9. Recommendation

The study is recommended to the government to increase the level of education of the mother; also, the study is recommended to increase the hospital delivery that may reduce the immunization coverage in Somalia. The study also recommended the government and the NGOs working in Somalia, to expand the program of immunization.

Finally, further studies focusing on assessment factors associated with complete immunization coverage among children in aged 11-24 months with different study design to compare their outcomes are required to help improve the national and regional health of Somalia.

## Figures and Tables

**Table 1:** Sociodemographic characteristics.

Variable	Frequency	Percentage
1. Age		
11-14	111	31.1%
15-19	137	38.4%
20-24	109	30.5%
Total	357	100%
2. Education		
Nonformal	263.	73.7%
Primary	66.	18.5%
Secondary	17.	4.8%
Tertiary	11.	3.0%
Total	357	100%
3. Marital status		
Windowed	43.	12.%
Married	277.	77.60%
Divorce	37.	10.4%
Total	357	100%
4. Occupation		
Employed	80.	22.4%
Nonemployed	277.	77.6%
Total	357	100%
5. Family income		
50-100	72.	20.2%
110-150	55.	15.4%
160-210	89.	24.9%
220-600	141.	39.5%
Total	357	100%
6. Residence		
Hormar	32.	9.0%
Howl/Wadag	111.	31.1%
Wadajir	48.	13.4%
Garsor	94.	26.3%
Rural	72.	20.2
Total	357	100%
7. ANC attendedtime		
No visits	191	53.5%
Less than 4	128.	35.9%
4 and above	38.	10.6%
Total	357.	100%
8. Sex of child		
Male	145	40.6% 59.4%
Female	212.	100%
Total	357	
9. Birth order		
1-4	199.	55.7%
5-8	112.0	31.4%
9-15	46.	12.9%
Total	357	100%
Mean age (SD) years		
7.71 ± 5.87		

**Table 2:** Factors association the immunization coverage in Somalia.

	Immunization status		Total	*p* value
	Fully vaccinated	Not fully vaccinated		
Age of the child				
11-14	26.0	158.0	184.0	0.14700000
15-19	26.0	59.0	85.0	
20-24	18	70.0	88	
Total	70.0	287.0	357	
Occupation of the mother				
Employed	11.0	69.0	80.0	0.134094
Nonemployed	59.0	218.0	277.0	
Total	70.0	287.0	357	
Distance to health facility				
Below 1 km	10	58.0	68.0	0.026533∗∗
1–2 km	31	71.0	102	
2.1–5 km	18	91	109	
5.1–10 km	6	30	36	
More than >10	5	37	42	
Total	70	287	357	
Sex of child				
Male	25.0	120.0	145	0.351655
Female	45	167	212.	
Total	70	287.	357	
Marital status of the mother				
Married	52.0	225.0	277	0.707352
Divorce	9	28	37.	
Windowed	9.0	34	43	
Total	70	287	357	
Antenatal care for the mother				
No visits	38.0	153.0	191	0.456024
Less than 4	22.0	106.0	128	
4 and above	10.0	28.0	38	
Total	70.0	287.0	357	
Level of education of the mother				
Nonformal	35.0	228.0	263	0.000001∗∗
Primary	16.0	50.0	66	
Secondary	11	6	17	
Tertiary	8.	3	11	
Total	70	287	357	
Place of residence of the participants				
Hormar	9	23	32	0.091344
Howl/Wadag	18	93	121	
Wadajir	11	37	48	
Garsor	24	70	94	
Rural	8	64	72	
Total	70	287	357	
Place of delivery of the baby				
Home	10	162	172	0.000001∗∗
Hospital	60	125	185	
Total	70	287	357	
Birth order of the baby				
1-4	40	159	199	0.916631
5-8	22	90	121	
9-15	8	38	46	
Total	70	287	357	

**Table 3:** Predictors of full immunization coverage among respondents.

Variables	Crude odd ratio (COR)	*p* value
1. Place of delivery of the baby		
Home (ref)	1	
Hospital	7.375712 (3.43-15.824)	0.000001
2. Level of education of the mother		
Nonformal (ref)	1	
Primary	18.440 (4.0183-84.6212)	0.000177
Secondary	10.850335 (2.22036-53.022615)	0.003225
Tertiary	1.872193 (0.290881-12.049984)	0.509176
3 distance to health facility		
1 km(ref)	1	
1–2 km	1.316226 (0.363302-4.768624)	0.675691
2.1–5 km	0.550842 (0.172230-1.761755)	0.314769
5.1–10 km	1.000238 (0.301582-3.317421)	0.999690
More than >10	0.729598 (0.179925-2.958524)	0.658943

## Data Availability

The original data from this research is available in SPSS and excel spreadsheet, and it will be sent on request.
